# Proteome stability analysis of snap frozen, RNAlater preserved, and formalin-fixed paraffin-embedded human colon mucosal biopsies

**DOI:** 10.1016/j.dib.2016.01.061

**Published:** 2016-02-06

**Authors:** Tue Bjerg Bennike, Kenneth Kastaniegaard, Simona Padurariu, Michael Gaihede, Svend Birkelund, Vibeke Andersen, Allan Stensballe

**Affiliations:** a Research Unit for Molecular Diagnostic and Clinical Research, Hospital of Southern Jutland, Aabenraa, Denmark; b Department of Health Science and Technology, Aalborg University, Aalborg, Denmark; c Department of Otolaryngology, Head and Neck Surgery, Aalborg University Hospital, Aalborg, Denmark; d Department of Clinical Medicine, Aalborg University, Aalborg, Denmark; e Institute of Regional Health Research-Center Soenderjylland, University of Southern Denmark, Odense, Denmark.

**Keywords:** Human, Colon, Mucosa, RNAlater, FFPE, Snap-frozen, Stability, LC–MS, Proteomics

## Abstract

Large repositories of well characterized RNAlater preserved samples and formalin-fixed, paraffin-embedded samples have been generated worldwide. However, the impact on the proteome of the preservation methods remain poorly described. Therefore, we analyzed the impact on the proteome of preserving samples in RNAlater, and by formalin-fixation, paraffin-embedding on human soft tissue, using directly frozen samples as a control (“Comparing the proteome of snap frozen, RNAlater preserved, and formalin-fixed paraffin-embedded human tissue samples” [1]). We here report the data from the analysis. The comparative analysis was performed on 24 colon mucosa biopsies, extracted from the sigmoideum of two gastroenterologically healthy participants for the purpose of this study. A set of biopsies were additionally stored for 30 min at room temperature prior to formalin-fixation. The samples were analyzed by high throughput gel free quantitative proteomics. The MS proteomics data have been deposited to the ProteomeXchange Consortium via the PRIDE partner repository with the dataset identifier PRIDE: PXD002029.

**Specifications Table**

**Table:** 

Subject area	Biology
More specific subject area	Analysis of human colon tissue proteome stability, using different preservation methods.
Type of data	Raw files and text/excel files
How data was acquired	Data was acquired by bottom-up mass spectrometry on a Q Exactive plus (Thermo Scientific, Waltham, MA, USA) platform. The data was processed with MaxQuant.
Data format	Raw and Analyzed data.
Experimental factors	Following extraction, the human samples were preserved by direct freezing with liquid nitrogen, preserved in RNAlater, immediately formalin-fixed, paraffin-embedded, or stored for 30 min and formalin-fixed, paraffin-embedded.
Experimental features	The solubilized proteins were extracted and digested using a modified filter aided sample preparation protocol with trypsin. The peptide material was purified by ethyl-acetate phase inversion and analyzed by electrospray ionization liquid chromatography mass spectrometry. The raw-files were processed in the MaxQuant program package.
Data source location	Laboratory of Medical Mass Spectrometry, Department of Health Science and Technology, Aalborg University, Denmark.
Data accessibility	Data is within this article. MS proteomics data and search result files have been deposited to the ProteomeXchange Consortium via the PRIDE partner repository with the dataset identifier PRIDE: PXD002029.

**Value of the data**

• The dataset allows for assessing the impact of the commonly used sample preservation methods direct-freezing, RNAlater preservation, and formalin-fixed, paraffin-embedded, on protein identifications, post-translational modifications, and protein abundances.
• The dataset allows for assessing the impact on the proteome of delaying tissue stabilization for 30 min.
• The dataset can be used to build enhanced spectral libraries, e.g. for data-independent acquisition methods and identify colon-specific proteins due to the well characterized origin and processing of the samples.
• Our results demonstrate the feasibility in preserving tissue samples in RNAlater, enabling coupled proteomics, transcriptomics, and genomic studies from uniformly preserved samples.

## 1 Data

Human colon mucosal biopsies were extracted and either directly frozen with liquid nitrogen (DF), preserved in RNAlater, or formalin-fixed, paraffin-embedded (iFFPE). One set of biopsies were stored for 30 min (sFFPE) at ambient temperature before stabilization in formalin.

The biopsies were analyzed by label-free proteomics, resulting in Thermo MS raw-files (Table 1) and text-based protein identification and quantification result-files (Table 2). All files have been deposited to the ProteomeXchange Consortium via the PRIDE partner repository and can be downloaded by using the dataset identifier PXD002029 [2], [3]. Additionally, the full list of identified proteins in the colon biopsies (< 1% false discovery rate) has been submitted (Supplementary Table 1). The full analysis and interpretation of the data has been published in EuPa Open Proteomics 2015 [1].

## 2 Experimental design, materials and methods

### 2.1 Collection of sample material

Colon mucosal biopsies were sampled from the sigmoideum of two gastroenterologically healthy participants, by endoscopy at Hospital of Southern Jutland, Aabenraa, Denmark [4]. Twelve biopsies were extracted from each person approximately 40 cm from the anus. All biopsies had an approximate size of 1−2 mm^3^, and the biopsies were preserved by four different methods: (1) directly frozen biopsies (DF) were immediately transferred to individual cryotubes and snap-frozen with liquid nitrogen followed by storage at −80 °C for one month prior to sample processing and proteome analysis. (2) RNAlater biopsies were immediately transferred to individual cryotubes prefilled with 0.5 mL RNAlater (Life Technologies, Carlsbad, CA, USA), stored at room temperature for 24 h followed by storage at −80 °C for one month prior to sample processing and proteome analysis. FFPE biopsies were placed in preparation cartridges and either immediately (iFFPE) stabilized in 4% formalin, or stored for 30 min (sFFPE) at ambient temperature before stabilization with 4% formalin. Paraffin embedding was performed after one week at the Department of Pathology, Aalborg University Hospital, Denmark, according to current standards. The FFPE prepared samples were subsequently stored for three weeks at room temperature prior to sample processing and proteome analysis.

The project was approved by The Regional Scientific Ethical Committee (S-20120204) and the Danish Data Protection Agency (2008-58-035), and all participants had given informed consent to participate.

### 2.2 Sample preparation

We utilized a modified FASP tryptic protein digestion protocol for the sample preparation to facilitate surfactant removal [7], [8], [9], [10], [11], [12], [13], [14], [15].

RNAlater and DF preserved samples were homogenized in 0.5 mL lysis buffer (12 mM sodium deoxycholate, 12 mM sodium dodecyl sulfate (SDC) in 300 mM Tris/HCl, pH 9.0) with steel beads, using a Bullet Blender Gold power-setting 10 for 5 min (Next Advance Inc., Averill Park, NY, USA). The homogenized samples were incubated at 95 °C for 10 min and sonicated for 10 min.

FFPE tissues were extracted using a scalpel, deparaffinized and rehydrated by washing in xylene (3×), and in 100% ethanol (2×), 96% ethanol (2×), 70% ethanol (2×), water. The samples were homogenized in 0.5 mL lysis buffer with steel beads, using a Bullet Blender Gold power-setting 10 for 5 min (Next Advance Inc., Averill Park, NY, USA). The homogenized samples were incubated at 95 °C for 60 min, and the samples were sonicated for 10 min.

Total protein concentration was determined by BCA and absorbance at 280 nm (A280) using a NanoDrop 1000 UV–vis Spectrophotometer (Thermo Scientific, Waltham, MA, USA). A volume corresponding to 100 µg protein was transferred to YM-30 kDa spin filters for protein digestion (Millipore, Billerica, MA, USA) and centrifuged. All centrifugation steps were performed at 14,000*g* for 15 min at 4 °C. Protein disulfide bonds were reduced with 12 mM tris(2-carboxyethyl)phosphine (Thermo Scientific, Waltham, MA, USA) for 30 min at 37 °C, and alkylate with 50 mM chloroacetamide (Sigma-Aldrich, St. Louis, MO, USA) for 20 min at 37 °C, and centrifuged after each step. The reducing and alkylating agents were dissolved in 120 mM SDC in 50 mM triethylammonium bicarbonate (TEAB), pH 8.5. In preparation for digestion, 400 µL digestion buffer (12 mM SDC in 50 mM TEAB) was added to the spin filter and centrifuged. A 1:50 (w/w) trypsin:protein ratio dissolved in 50 µL digestion buffer was added to the spin filter, and the samples were digested overnight at 37 °C. The flow-through containing the peptides was retrieved by addition of 50 µL digestion buffer and centrifugation. SDC was removed by performing phase separations with 3:1 (v/v) ethyl acetate:sample, acidified by addition of FA to a final concentration of 0.5%. Total phase separation was achieved by 2 min agitation followed by centrifugation. The aqueous phase was collected and vacuum centrifuged overnight and the dry peptide product was stored at −80 °C until time of analysis.

### 2.3 Mass spectrometry analysis

The samples were resuspended in 2% ACN, 0.1% FA, briefly sonicated, and 5 µg total peptide material was analyzed per LC-MS analysis, in a random sample order [16]. The samples were analyzed using a UPLC-nanoESI MS/MS setup with an UltiMate 3000 UHPLC system (Dionex, Sunnyvale, CA, USA) upgraded with a RSLC nanopump module. The system was coupled online with an emitter for nanospray ionization (New objective picotip 360-20-10) to a Q Exactive Plus mass spectrometer (Thermo Scientific, Waltham, USA). The peptide material was loaded onto a 2 cm trapping reversed phase Acclaim PepMap RSLC C18 column (Dionex), and separated using an analytical 50 cm reversed phase Acclaim PepMap RSLC C18 column (Dionex). Both columns were kept at 40 °C. The sample was eluted with a gradient of 96% solvent A (0.1% FA) and 4% solvent B (0.1% FA in ACN), which was increased to 30% solvent B on a 180 min ramp gradient at a constant flow rate of 300 nL/min. The mass spectrometer was operated in positive mode, selecting up to 12 precursor ions with a mass window of *m*/*z* 1.6 based on highest intensity for HCD fragmenting, at a normalized collision energy of 27. Selected precursors were dynamically excluded for fragmentation for 30 s.

### 2.4 Protein identification and quantitation

A label-free quantative analysis was performed in MaxQuant 1.5.1.2. The raw-files were searched against the Uniprot Homo sapiens reference proteome (UP000005640, last modified 2015-01-16, protein count 68,015) [17], [18]. All standard settings were employed with carbamidomethyl (C) as a static modification and protein N-terminal acetylation, deamidation (NQ), oxidation (M) and peptide N-terminal formylation as variable modifications [5], [6]. All reverse hits and proteins tagged as contaminants were removed from further analysis, and all proteins are reported below 1% false discovery rate.

## Figures and Tables

**Table 1:** Description of raw file-names in the ProteomeXchange repository PXD002029. DF: directly frozen; iFFPE: immediate formalin-fixed, paraffin-embedded; sFFPE: stored for 30 min prior to formalin-fixed, paraffin-embedded. Each file represents an analysis of one biopsy.

**Raw-files**	**Samples type**	**Participant**	**Protocol**
Colon_ParticipantA_DF_1.raw	Human colon mucosa	A	DF
Colon_ParticipantA_DF_2.raw	Human colon mucosa	A	DF
Colon_ParticipantA_DF_3.raw	Human colon mucosa	A	DF
Colon_ParticipantA_RNAlater_1.raw	Human colon mucosa	A	RNAlater
Colon_ParticipantA_RNAlater_2.raw	Human colon mucosa	A	RNAlater
Colon_ParticipantA_RNAlater_3.raw	Human colon mucosa	A	RNAlater
Colon_ParticipantA_iFFPE_1.raw	Human colon mucosa	A	iFFPE
Colon_ParticipantA_iFFPE_2.raw	Human colon mucosa	A	iFFPE
Colon_ParticipantA_iFFPE_3.raw	Human colon mucosa	A	iFFPE
Colon_ParticipantA_sFFPE_1.raw	Human colon mucosa	A	sFFPE
Colon_ParticipantA_sFFPE_2.raw	Human colon mucosa	A	sFFPE
Colon_ParticipantA_sFFPE_3.raw	Human colon mucosa	A	sFFPE
Colon_ParticipantB_DF_1.raw	Human colon mucosa	B	DF
Colon_ParticipantB_DF_2.raw	Human colon mucosa	B	DF
Colon_ParticipantB_DF_3.raw	Human colon mucosa	B	DF
Colon_ParticipantB_RNAlater_1.raw	Human colon mucosa	B	RNAlater
Colon_ParticipantB_RNAlater_2.raw	Human colon mucosa	B	RNAlater
Colon_ParticipantB_RNAlater_3.raw	Human colon mucosa	B	RNAlater
Colon_ParticipantB_iFFPE_1.raw	Human colon mucosa	B	iFFPE
Colon_ParticipantB_iFFPE_2.raw	Human colon mucosa	B	iFFPE
Colon_ParticipantB_iFFPE_3.raw	Human colon mucosa	B	iFFPE
Colon_ParticipantB_sFFPE_1.raw	Human colon mucosa	B	sFFPE
Colon_ParticipantB_sFFPE_2.raw	Human colon mucosa	B	sFFPE
Colon_ParticipantB_sFFPE_3.raw	Human colon mucosa	B	sFFPE

**Table 2:** Description of other file-names in the ProteomeXchange repository PXD002029.

**Filename**	**Description**
biopsies_combined_search.zip	The zipped MaxQuant result folder “combined”, containing the result of the label-free analysis as well as additional information.
uniprot-proteome_3AUP000005640_iso.fasta	Uniprot human reference proteome database used to identify proteins in the dataset.
FASTA info.txt	Additional information about the utilized Uniprot database, e.g. protein count, date ect.

## Footnotes

Appendix A Supplementary data associated with this article can be found in the online version at 10.1016/j.dib.2016.01.061.
